# Phosphate Elimination in Emulsified Meat Products: Impact of Protein-Based Ingredients on Quality Characteristics

**DOI:** 10.3390/foods10040882

**Published:** 2021-04-17

**Authors:** Olivier Goemaere, Seline Glorieux, Marlies Govaert, Liselot Steen, Ilse Fraeye

**Affiliations:** Research Group for Technology and Quality of Animal Products, Department of Microbial and Molecular Systems, Leuven Food Science and Nutrition Research Centre (LFoRCe), KU Leuven Ghent Technology Campus, Gebroeders De Smetstraat 1, B-9000 Gent, Belgium; olivier.goemaere@kuleuven.be (O.G.); seline.glorieux@tradelio.eu (S.G.); marliesgovaert@hotmail.com (M.G.); Liselot.Steen@solina-group.eu (L.S.)

**Keywords:** phosphate elimination, emulsified meat products, proteins, standardized meat matrix

## Abstract

The addition of phosphates to meat products improves the emulsifying and gelling properties of meat proteins, in turn enhancing overall product quality. The current market trend towards additive-free products and the health issues related to phosphate challenge the industry to develop phosphate-free meat products. The aim of this study was to evaluate the potential of seven protein-based ingredients (pea, blood plasma, gelatin, soy, whey, egg, and potato) to remediate quality losses of emulsified meat products (cooked sausages) upon phosphate elimination. First, the intrinsic gelling and emulsifying characteristics of the proteins were assessed. Next, the proteins were added to phosphate-free sausages, of which quality characteristics during production (viscoelastic behavior and emulsion stability) and of the final products (texture, cooking loss, and pH) were screened. Blood plasma and soy were superior in phosphate-free cooked sausages, as no significant differences in hardness, cooking yield, or stability were found compared to phosphate-containing sausages. Egg and pea also improved the previously mentioned quality characteristics of phosphate-free sausages, although to a lesser extent. These insights could not entirely be explained based on the intrinsic gelling and emulsifying capacity of the respective proteins. This indicated the importance of a well-defined standardized meat matrix to determine the potential of alternative proteins in meat products.

## 1. Introduction

Food phosphates exist in different types (mono-, di-, tri-, and polyphosphates) and are often used in the meat industry due to their impact on pH, chelation, ionic strength, and antibacterial activity. They fulfill several functional properties in meat products such as a good buffering capacity (monophosphates) and the ability to dissociate the actomyosin complex of meat (diphosphates) and activate the meat proteins by chelating the protein-bound Mg^2+^ and Ca^2+^, leading to increased solubilization of the meat proteins and depolymerization of the thick and thin filaments (tri- and polyphosphates) [1,2]. Due to these effects, meat proteins can maximally exert their emulsifying and gelling properties, which are very important with regard to water holding capacity (WHC) and fat emulsification. In addition, most di- or polyphosphates contribute to an increase in pH or ionic strength, respectively. Both effects result in increased electrostatic repulsion and consequently more space to bind water and fat between the meat proteins, which again contributes to increased water and fat stabilization. The different phosphate types (or blends) in meat products can be added to a maximum amount of 0.5% (expressed as P_2_O_5_) according to European legislation [3]. However, in a former study of Ritz, et al. [4], an association was found between a high intake of phosphate additives and cardiovascular morbidity and mortality. This health issue was already recognized for chronic kidney disease patients, but questions arose with regard to the general population. The EFSA Panel on Food Additives and Flavorings further investigated the matter and provided a scientific opinion re-evaluating the safety of phosphates as food additives in 2019. They considered phosphates to be of low acute oral toxicity, and there was no concern with respect to genotoxicity and carcinogenicity. Furthermore, the Panel considered an acceptable daily intake (ADI) of 40 mg/kg body weight per day. However, this ADI does not apply to humans with a reduction in renal function. Ten percent of the general population might have chronic kidney disease with reduced renal function and they may not tolerate the proposed ADI [5].

In a recent study, it was shown that the current amount of P_2_O_5_ added to emulsified meat products (cooked sausages) can be strongly reduced with minimal loss in product quality [6]. Nevertheless, the market trend towards additive-free products [2,7] and the negative effect of phosphates on human health for certain population groups justify attempts to develop phosphate-free emulsified meat products. Unfortunately, phosphate elimination results in decreased meat protein functionality, which causes quality defects such as compromised water and fat stabilization [6]. Therefore, alternative ingredients or even innovative technologies are needed to compensate for this functionality loss. These include pH improving ingredients, starches, hydrocolloids, or the use of high-pressure technology [2,8,9,10,11]. Additionally, proteins, from both animal and vegetable sources, can act as enhancers to compensate for the loss of functionalized meat proteins due to phosphate elimination in meat products. This is mainly related to their gelling and emulsification properties. They have already been proven useful to boost the quality characteristics of meat products related to water and fat binding properties, gel network formation, texture, and/or sensorial properties. In this respect, they have been successfully deployed as fat-replacers, processing aids of low-cost meat products, and substitutes for meat proteins [12,13,14,15,16,17,18,19,20].

However, only a limited number of studies aimed to investigate the opportunities of reduction/replacement of phosphate in meat products [11], especially with regard to the use of alternative proteins. Hurtado, et al. [21] concluded that porcine blood plasma was a useful functional ingredient to replace phosphate and caseinate in frankfurters. Pereira, et al. [22] stated that the addition of collagen fibers improved cooking yield and hardness in phosphate-free sausages. Enhancement of water holding capacity, sensorial attributes, color, and microbial stability could also be achieved by replacing phosphates with a purified beef collagen powder in injected beef strip loins [23]. Furthermore, Muguruma, et al. [24] stated that the addition of biopolymers containing soybean and milk proteins may permit a reduction in phosphate content without a loss of the texture of chicken sausages.

In summary, alternative proteins have been proven to function as functional ingredients in different meat systems on account of their gelling and emulsifying capacities. In contrast, the more specific ability to act as a phosphate replacer, in order to manage the loss of functionalized meat proteins, has only been studied for a limited number of proteins. Furthermore, standardized comparison between proteins remains difficult, because these surveys were conducted on different meat matrices (difference in meat product class, composition of recipes, processing conditions) and different analyzing techniques were applied, making it impossible to identify the most promising protein. Xiong [25] stated that a valid comparison between proteins is only possible if the screening is made under identical processing and storage conditions. Therefore, the aim of this study was to evaluate the potential of seven protein-based ingredients, from both animal and vegetable sources, to counter the loss of quality due to phosphate elimination in emulsified meat products (cooked sausages). In the first stage, the intrinsic protein characteristics (gelation and emulsification potential), related to improving the quality of meat products, are studied. In the second stage, the ability of the proteins to enhance the properties of phosphate-free cooked sausages (viscoelastic and textural properties, emulsion stability, cooking loss, and pH) is evaluated. This research is of important industrial relevance, since phosphate elimination in emulsified meat products will decrease potential health concerns and is a promising step towards clean-label products.

## 2. Materials and Methods

### 2.1. Determination of the Intrinsic Characteristics of Selected Proteins

The proteins discussed in this study are egg white (Pulviver), pea (Nutralys), potato (KMC), soy concentrate (Pulviver), blood plasma (Veos), gelatin (Rousselot), and whey (Caldic) protein. Proteins were selected based on their industrial relevance. In addition, a balanced distribution between animal and vegetable proteins was envisioned. In order to learn more about their intrinsic properties related to enhancing meat product quality, their gelation and emulsification potential were studied in a watery environment. It is well described that the salt level and acidity of a medium have an important impact on protein characteristics [12,26,27]. In order to create an aqueous medium that reflects the composition of emulsified meat products, proteins were suspended in a 0.05 M Na phosphate buffer (pH = 6) containing 3.5% NaCl and stirred (800 rpm) for 105 min at room temperature before analysis. The applied protein concentration was dependent on the screening technique used, as described below. The concentration of salt corresponds to its quantity in the cooked sausage model (see Section 2.2) expressed in the water phase, and the pH value is in the range of common meat products.

#### 2.1.1. Gelation Potential

The gelation properties of the proteins were determined through rheological measurements using an AR2000ex stress-controlled rheometer (TA instruments, New Castle, DE, USA) equipped with a 40-mm parallel plate system. A crosshatched upper plate and a lower plate were used to prevent slippage of the sample. The gap was set at 500 μm. The AR2000ex was supplemented with an efficient Peltier temperature control system and an upper heated plate (TA Instruments) to control the sample temperatures accurately. Temperature sweeps were conducted to investigate structural changes of the protein suspensions (4.5% protein) during a heating and cooling process, representative of the manufacturing process of emulsified meat products. The following profile was applied: (1) a heating step from 20 to 76 °C at a constant heating rate of 2 °C/min; (2) an isothermal heating step at 76 °C for 3 min; (3) a cooling step from 76 to 20 °C at a constant cooling rate of 2 °C/min. Oscillation measurements during the entire process were performed at a fixed frequency of 1 Hz and a strain of 0.025, a value found to be within the linear viscoelastic region based on preliminary experiments. The storage modulus (G’) and phase angle (δ, with δ of 90° representing a fully viscous material and δ of 0° representing a fully elastic material) at the end of the isothermal heating step and cooling step (G’_76 °C,suspension_, δ_76 °C,suspension_ and G’_end_,_suspension_, δ_end_,_suspension_, respectively) were derived from the temperature sweep profiles using the software (Rheology Advantage Data Analysis, v. 5.7.0, TA Instruments). All G’-values are expressed logarithmically. Protein suspensions were made in duplicate for each protein, and determination of the gelling potential was performed in duplicate per suspension.

#### 2.1.2. Emulsification

The emulsifying properties of the proteins were screened according to the procedure described by Steen, et al. [28], which was based upon the turbidimetric method of Pearce and Kinsella [29]. Emulsions were prepared by mixing 2.0 mL sunflower oil and 8.0 mL protein suspensions (0.15% protein) for 1 min at a speed of 12,000 rpm and room temperature using an Ultra-Turrax homogenizer (model T25, IKA-Werke GmbH, Staufen, Germany). Immediately and 10 min after emulsion formation, 50 μL of the emulsion was taken and diluted with 5 mL of 0.1% sodium dodecyl sulfate (SDS) solution. Absorbance values were measured at 500 nm (A_500_) and used to calculate the emulsifying activity index (EAI, m^2^/g) according to the equations below.

(1) $$\mathrm{EAI}\;(m^{2}/g)=\frac{2xTxF}{\varphi \;C}$$

(2) $$T=\frac{2.303xA500}{L}$$

where A_500_ represents the absorbance at 500 nm, *L* the light path length (*L* = 0.01 m), *φ* the volume fraction (v/v) of the dispersed phase (*φ* = 0.20), *C* the protein concentration (*C* = 1500 g/m^3^) before emulsification, *T* the turbidity, and *F* the dilution factor (*F* = 100). The emulsion activity index immediately after emulsion formation is represented by the abbreviation EAI0. Emulsion stability (ES) was the percentage of emulsion turbidity remaining after 10 min. Emulsions were made in duplicate for each protein, and determination of the emulsifying properties was performed in duplicate for each emulsion.

### 2.2. Manufacturing of Cooked Sausage

Cooked sausages were prepared in the pilot plant of the research group “Technology and Quality of Animal Products” (KU Leuven Technology Campus Gent, Belgium). Raw materials (pork shoulder and pork backfat) were obtained from a local industrial meat supplier (De Lausnay Rene bvba, Destelbergen, Belgium), chopped, homogenized to generate one batch, vacuum-packed, and stored at –18 °C until preparation. Cooked sausages contained pork shoulder (35/100 g), pork backfat (35/100 g), and ice (30/100 g), together with nitrite curing salt (1.5/100 g), sugar (0.5/100 g), white pepper (0.2/100 g), foil (0.05/100 g), ascorbic acid (0.05/100 g), glutamate (0.05/100 g), coriander (0.025/100 g), and cardamom (0.025 g/100 g). All non-meat ingredients were purchased from Solina Group (Eke-Nazareth, Belgium). Ingredients were calculated relative to the total mass of meat raw materials (pork shoulder and pork backfat) and ice. First, a standardized reference treatment was prepared, containing 0.32/100 g tetrasodium pyrophosphate (TSPP) (Solina Group, Eke-Nazareth, Belgium), which is equal to 0.171% P_2_O_5_, a standard amount used in the meat industry for emulsified meat products. The reference containing phosphate will be referred to as M+TSPP. Secondly, TSPP was eliminated and standardized phosphate-free cooked sausages were prepared. These cooked sausages will be referred to as M-TSPP. Finally, the seven above-described protein-based ingredients were added to the phosphate-free treatment. All proteins were added in a mass fraction of 2/100 g, a commonly used dosage [17]. Proteins were calculated relative to the total mass of meat raw materials (pork shoulder and pork backfat) and ice. These phosphate-free treatments containing protein-based ingredients will be referred to as M-TSPP+“corresponding protein source”. During manufacturing of the M+TSPP preparations, the raw lean meat was first pre-chopped together with ice, salt, and TSPP in a bowl cutter for 7 min and 30 s (Stephan cutter UM12, Hameln, Germany), corresponding to a final temperature of 5 °C. Next, the pork backfat was added to the meat batter together with the remaining food ingredients. The total mass was ground under vacuum for 4 min and 30 s to obtain a homogenous batter. The temperature did not exceed 14 °C during processing to avoid protein denaturation and fat coalescence. Phosphate-free sausages were prepared the same way, but without the addition of TSPP. When phosphate-free sausages containing protein-based ingredients were prepared, these proteins were added during the first grinding step of the manufacturing process instead of TSPP. Part of the batter, prepared in duplicate per treatment, was immediately analyzed for dynamic viscoelastic properties (Section 2.3) and emulsion stability (Section 2.4). In order to standardize the cooking process, the remainder of the batter was filled into cans of standardized dimensions (diameter 7 cm, height 5 cm, mass ± 250 g, Crown Verpakking België NV, Hoboken, Belgium), cooked at 76 °C (core temperature 72 °C) for 90 min in a cooking chamber (Rational Climaplus Combi CPC 61, Claes Machines, Paal, Belgium) and finally cooled to 4 °C. The resulting meat products, cooked in cans, served as a model product for cooked sausage and will be referred to as “cooked sausages”. Each treatment, including the reference products with or without TSPP, was manufactured in duplicate. One week after the manufacturing process, three sausages per replicate were analyzed for cooking loss (Section 2.5), pH (Section 2.6), and textural properties (Section 2.7). The number of measuring points is described in the respective analyses below.

### 2.3. Dynamic Viscoelastic Properties

The dynamic viscoelastic properties of the batters were analyzed using the same equipment as described in Section 2.1.1. The gap was set at 1000 μm for both rheological procedures described below (stress sweep and temperature sweeps).

Stress sweeps were conducted at a temperature of 13 °C, between 0.1 and 1000 Pa, and at a fixed frequency of 1 Hz to determine the linear viscoelastic region (LVR). Hereby, parameters G’, G” (storage and loss modulus respectively), and δ were directly obtained from the software. The complex modulus (G*), representing the materials’ overall rigidity or resistance to deformation, was calculated by the following formula,

(3) $$G*=\sqrt{G^{\text{′}2}+G^{\text{″}2}}$$

The LVR represents the stress range within which G* (and thus G’,G”) is independent of the imposed stress amplitude and is determined according to Glorieux, Goemaere, Steen and Fraeye [6]. LVR is determined in duplo per replicate of each treatment and referred to as LVR_batter_. Furthermore, the corresponding G*_batter_, expressed logarithmically, and δ_batter_ within the LVR are reported.

Temperature sweeps were conducted to investigate the impact of phosphate elimination and use of alternative proteins on the structure formation of meat batters during a heating and cooling procedure, representative of the manufacturing process of cooked sausages. Similar profiles and conditions were applied as described in Section 2.1.1., except for the initial (before heating) and final (end of cooling) temperatures, which were both set at 13 °C in accordance with the final temperature of the raw batter at the end of the cutter process. The parameters G’ and δ at the end of the isothermal heating step and cooling step (G’_76°C,batter_, δ_76°C, batter_ and G’_end_,_batter_ δ_end_,_batter_, respectively) were derived from the temperature sweep profiles using the software. G’-values are expressed logarithmically. All rheological parameters (G’_76°C,batter_, δ_76°C,batter_, G’_end_,_batter_, δ_end_,_batter)_ were determined in duplo per replicate of each treatment.

### 2.4. Emulsion Stability

Emulsion stability of the meat batter was determined immediately after the grinding process, according to Glorieux, Goemaere, Steen and Fraeye [6] with slight modifications. Summarized, emulsion stability is expressed as drip loss upon heating (30 min, 70 °C) and centrifugation at 4230× *g* (6000 rpm in a rotor Cat. No. 1620 A, Hettich, Germany) at 25 °C for 3 min, of a pre-weighed amount of raw batter. The percentage of total expressible fluid (TEF) was expressed as follows:

(4) $$\mathrm{TEF}\;(\%)=\frac{drip\;loss\;meat\;batter}{initial\;weight\;meat\;batter}\times 100$$

Furthermore, the relative amount of water, next to the fat in the drip, was determined. Therefore drip loss after centrifugation was weighed before and after drying in an oven (Typ U 40, Memmert, Germany) for 24 h. The relative amount of water in the drip loss was expressed as follows:

(5) $$\mathrm{Relative}\;\mathrm{amount}\;\mathrm{of}\;H_{2}O\;\mathrm{in}\;\mathrm{drip},\;(\%)=\frac{drip\;before\;drying-drip\;after\;drying}{drip\;before\;drying}\times 100$$

TEF and Relative amount of H_2_O in drip were determined six times per replicate of each treatment.

### 2.5. Cooking Loss

Cooking loss (CL) of the cooked sausages of each treatment was measured according to Glorieux, Goemaere, Steen and Fraeye [6]. CL was calculated as follows:

(6) $$\mathrm{CL}\;(\%)=\frac{drip\;loss\;sausage}{initial\;weight\;sausage}\times 100$$

Measurements were determined in triplicate per replicate of each treatment.

### 2.6. pH Measurement

The pH of the cooked sausages was measured three times on three different sausages (nine measurements) per replicate of each treatment, according to the methods described in Glorieux, Goemaere, Steen and Fraeye [6].

### 2.7. Texture

The hardness of the cooked sausages was analyzed using a Lloyd Texture Analyzer (Model LF plus, Lloyd Instruments, Bognor Regis, UK) and expressed as the maximum force (N) to penetrate the sample, as described in Glorieux, Goemaere, Steen and Fraeye [6]. Per replicate of each treatment, hardness was measured three times on three different sausages (nine measurements).

### 2.8. Statistical Analysis

Results are expressed as mean ± standard deviation. All results were evaluated by one-way ANOVA. A Tukey’s post hoc test was performed with a significance level of *p* < 0.05 to identify significant differences. Statistical analysis was performed using the software IBM SPSS Statistics 25 (IBM, Armonk, NY, USA).

## 3. Results and Discussion

### 3.1. Intrinsic Characteristics of Selected Proteins

Screening of the intrinsic characteristics of functional ingredients is often executed in watery media. It is a rather quick and easy method to evaluate ingredient functionality that requests no specific and often expensive process equipment to imitate industrial food products. Moreover, it can provide a broad view of the application potential of the ingredients in several food products. Food proteins are mainly applied in meat products in relation to their gelling and emulsifying properties, enabling them to improve overall meat product quality. Results regarding these intrinsic characteristics are described below in Section 3.1.1 and Section 3.1.2.

#### 3.1.1. Gelation Potential

**Figure 1:**
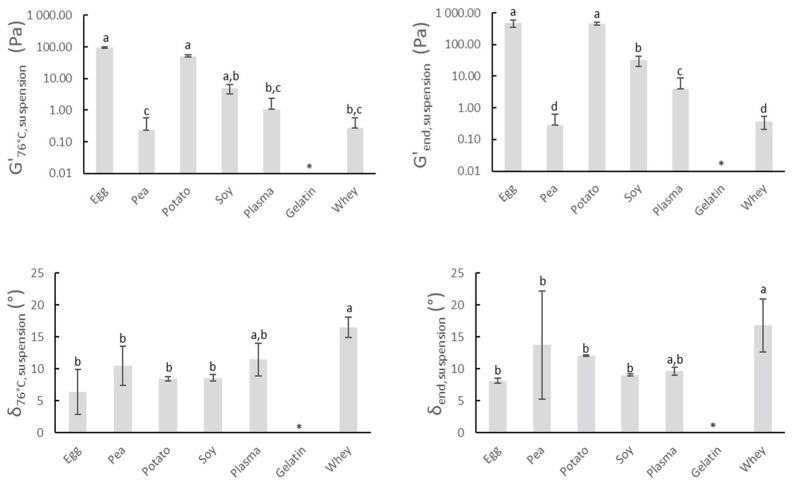
G’_76°C,suspension_ and δ_76°C,suspension_ represent the elasticity modulus and phase angle of protein suspensions at the end of the isothermal heating at 76 °C for 3 min. G’_end,suspension_ and δ_end,suspension_ represent the elasticity modulus and phase angle of protein suspensions after further cooling from 76 to 20 °C. (*) = no value of gelatin could be obtained. Mean values and standard deviations are presented (n = 4). Letters a–d: different letters indicate significant differences (*p* < 0.05) between different proteins.

The gelling characteristics of proteins are one of the key reasons they are applied for meat product improvement. Figure 1 shows the gelling properties of the proteins upon heating, with the exception of gelatin. Gelatin is a cold-gelling protein that solubilizes during heating [30] and can therefore only participate in gel network formation at sufficiently low temperatures. The critical temperature below which gelling can occur is dependent on gelatin concentration, cooling rate, and maturing temperature [31]. The applied thermal processing and used gelatin (concentration, source) did not allow the expression of the cold gelling character of gelatin. For all other protein suspensions, the heating and subsequent cooling process caused in general an overall increase in G’ and a decrease of δ. This suggests the formation of a gel-like structure and increased elastic behavior. G’_76°C,suspension_ and G’_end,suspension_ are highest for potato and egg white protein, indicating the strongest gelling potential of all screened proteins. The irreversible heat coagulation of egg white proteins involves the formation of spherical aggregates via hydrophobic interactions, which are further stiffened through sulfhydryl–disulfide reactions to finally give rise to a gel, which explains the rather high values of G’_76°C,suspension_. Furthermore, the G’-values of egg white protein still increase (G’_76°C,suspension_ vs. G’_end,suspension_) during cooling, which can be attributed to the numerous hydrogen bonds that are formed at lower temperatures [32]. The suspension of potato protein also exerted very good gelling properties upon heating. The low denaturation temperature of patatin, one of the main potato protein fractions, may be partially responsible for this. The denaturation temperature is roughly 20 °C lower compared to common food proteins as ovalbumin (egg) or soy glycinin [33,34]. Figure 1 reveals no significant difference between G_’76°C,suspension_ of the egg white protein and G’_76°C,suspension_ of soy concentrate, indicating good gelation characteristics of the latter. Pea proteins are mainly composed of globulins. Pea globulins are recognized for their lower gelling ability compared to their soy counterparts. This can also be observed in Figure 1, where G’_76°C,suspension_ and G’_end,suspension_ of pea proteins are significantly lower than the values of soy concentrate. The gelation of pea proteins appeared to be governed mainly by nonspecific interactions, whereas the involvement of disulfide bonds was reported for soy proteins [35]. Furthermore, high temperatures are required to induce the gelation of the pea proteins because of their high denaturation temperature (>85 °C) [36]. The applied thermal processing in this research was therefore not sufficient to obtain proper gelling of pea proteins. Whey protein suspensions start to form gels at concentrations higher than 80 mg whey protein/g H_2_O when heated above 75 °C [37]. The rather short heating time above 75 °C and applied concentration could therefore explain the somewhat low values of G’_76°C,suspension_, and G’_end,suspension_ of whey protein. Blood plasma also showed relatively low values of both G’_76°C,suspension_, and G’_end,suspension_ and is probably attributed to the same reasons as described for whey protein. Research stated that heating to 75 °C was a necessity to create strong gels from 4% *w*/*v* plasma protein solutions [38]. Other sources claimed that suspensions containing 4–5% blood plasma already tend to form firm and irreversible gels when temperatures over 70 °C are applied [39].

Based upon the gathered data and literature study, potato and egg white protein show the most potential for use in meat products. Their gelling properties may lead to a better structure formation of the meat gel and as a consequence to improved water binding or texture of the sausages.

#### 3.1.2. Emulsification

**Figure 2:**
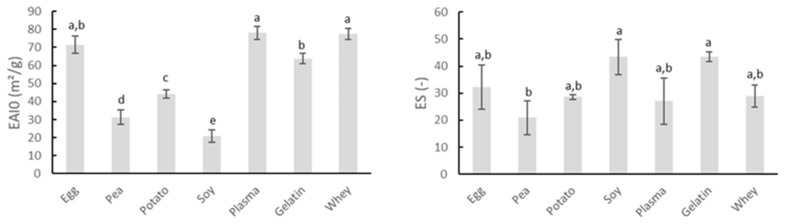
Comparison of emulsifying activity index immediately after emulsion formation (EAI0, m^2^/g) and emulsion stability (ES) of several protein sources. Mean values and standard deviations are presented (n = 4). Letters a–e: different letters indicate significant differences (*p* < 0.05) between different proteins.

In addition to their gelation potential, proteins are also of interest to the meat industry because of their ability to stabilize emulsions. The intrinsic emulsifying and emulsion-stabilizing properties of the proteins can be derived from Figure 2. The EAI0 indicates the area of interface stabilized per unit weight of protein (m^2^/g) and is associated with the ability of the protein to coat the water–oil interface immediately after emulsion formation. ES represents the percentage of emulsion turbidity remaining after 10 min and therefore refers to the ability of an emulsion to resist changes in its properties over time, e.g., droplet coalescence, creaming, and/or flocculation [28].

Gelatin possesses good emulsifying properties, as can be noticed by the high values of both EAI0 and ES in Figure 2. Gelatin is capable of reducing the surface tension of aqueous environments and forming the necessary identically charged film around the fat droplets of the dispersed phase. Therefore, the isoelectric point (IEP) is of great importance in the surface activity effects of the used gelatin [40]. The protein carried a net negative charge under the conditions in which this analysis was performed. Whey proteins are well-known for their ability to stabilize interfaces, explaining their great emulsifying properties, as seen in the present research [41]. Figure 2 also shows that blood plasma and egg white protein exerted excellent emulsifying properties. Research by Rodriguez Furlán, et al. [42] confirmed the good emulsifying properties of blood plasma. Yet, literature stated that ovalbumin, the major protein in egg white, may perform good emulsifying ability and stability under extreme acidic conditions, which is in contrast to the watery suspensions applied in this research, while under neutral and alkaline pH the stability of egg white emulsions was limited [43]. The emulsifying capacity of soy concentrate was rather limited, as indicated by the low value of EAI0 in Figure 2. The study of Amine, et al. [44] also indicated soy protein as a poor emulsifier for oil in water emulsions, based upon the measurement of oil droplet particle sizes. The same research presented potato protein as the better emulsifier compared to soy and pea proteins, as was the case in this study. Pea protein also exhibited poor emulsifying properties, as seen in Figure 2. Several studies concluded that pea proteins are usually inferior to traditional emulsifiers such as milk and egg proteins [45].

Results indicated the use of egg white protein, blood plasma, gelatin, or whey protein may be more beneficial in stabilizing meat emulsion regarding water and fat binding compared to the other screened proteins because of their high initial emulsion activity in combination with their good emulsion stability.

### 3.2. Impact of Seven Different Protein-Based Ingredients on the Quality Characteristics of Cooked Sausage

The results presented in the following sections deal with the impact of the selected proteins on several quality characteristics of phosphate-free sausage.

#### 3.2.1. Dynamic Viscoelastic Properties of Meat Batters Influenced by Protein Source

Stress sweeps were performed to study the structure of the raw meat batter immediately after the grinding process, prior to thermal processing. Data (Table 1, Stress sweeps) indicated that the LVR, the stress range in which the structure of the sample remains intact, significantly (*p* < 0.05) increased when TSPP was eliminated (M-TSPP) compared to the model preparation containing phosphate (M+TSPP). Since TSPP has the ability to dissociate the actomyosin complex [1], the M+TSPP batter was presumably more sensitive to external deformation. This is reflected in a significantly (*p* < 0.05) lower δ_batter_ value compared to the M-TSPP sample, the latter having more “solid-like” behavior. In parallel, the G*_batter_ of M-TSPP was significantly higher compared to M+TSPP, indicating that M-TSPP showed high resistance to deformation. A higher LVR_batter_, lower δ_batter_, and higher G*_batter_ as a result of phosphate elimination were also seen in our previous study [6].

The addition of protein-based ingredients to phosphate-free raw sausage batters did not affect the LVR_batter_ or G*_batter_ compared to the M-TSPP preparations, with the exception of the preparation with gelatin (M-TSPP+gelatin) and egg white protein (M-TSPP+egg). The addition of gelatin to phosphate-free raw sausage batter (M-TSPP+gelatin) significantly increased the G*_batter_, which can possibly be attributed to the cold gelling capacity of the protein [46]. Raw phosphate-free sausage batter containing 2% egg white protein (M-TSPP+egg) gave rise to a significantly lower LVR_batter_, and, at the same time, a remarkably high G*_batter_ compared to M-TSPP. An explanation of this striking observation is given in Appendix A.

**Figure 3:**
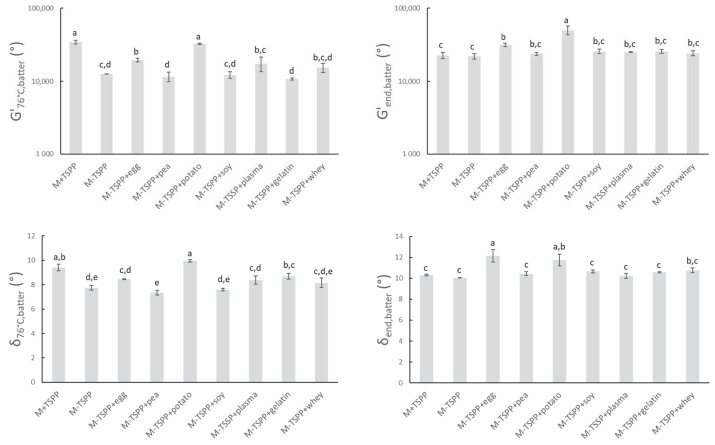
G’_76°C,batter_ and δ_76°C,batter_ represent the elasticity modulus and phase angle, respectively, of sausage batters during rheological measurements at the end of isothermal heating at 76 °C. G’_end,batter_ and δ_end,batter_ represent the elasticity modulus and phase angle of sausage batters after further cooling from 76 to 20 °C. Mean values and standard deviations are presented (n = 2). Abbreviations used: M (model) and TSPP (tetrasodium pyrophosphate). Letters a–e: different letters indicate significant differences (*p* < 0.05) between treatments.

To study the rheological properties of the sausage batters during thermal processing, all samples were subjected to a temperature sweep as described in Section 2.3. The heat causes the myofibrillar proteins to unfold and/or dissociate, followed by association and aggregation, resulting in a gelled system in which water and fat are entrapped [47,48]. The high G’_batter_-values in Figure 3 confirmed the formation of gel structures. δ_76°C,batter_ is lower than 10° for all batters, indicating a strong elastic behavior of the formed network. Significant differences in δ_76°C,batter_ between batters have little relevance.

Comparison between M+TSPP and M-TSPP indicated that the elimination of phosphate had an effect on the viscoelastic behavior of the meat batter during heating (G’_76°C,batter_ δ_76°C,batter_). Values of G’_76,batters_ revealed that phosphate elimination significantly reduced gel strength at the end of heating prior to cooling. On the other hand, upon subsequent cooling, no significant differences could be observed anymore between M+TSPP and M-TSPP (G’_end,batter_ and δ_end,batter_). The stronger increase of G’ during heating upon the addition of phosphates was possibly caused by conformation transitions, exposure of hydrophobic groups, and the formation of more disulfide bonds of the meat proteins [49]. In other words, TSPP promoted gelation, as it aids in the extraction of myofibrillar proteins that will subsequently aggregate and gel upon thermal processing [48,50,51,52]. However, Sun and Holley [48] also reported that it was possible that polyphosphates do not influence myofibrillar gel strength, as this is dependent on the applied protein source and preparation and gelation conditions that are used.

The strong gelling properties of potato protein nullified the drop in G’_76°C,batter_ due to phosphate elimination (M-TSPP+potato). A similar observation can be made when egg white protein (M-TSPP+egg) is used, although the total impact of phosphate elimination on G’_76,batters_ could not be compensated, since a significant difference in G’_76°C,batters_ between M+TSPP and M-TSPP+egg remained. Furthermore, G’_end,batter_ and δ_end,batter_ significantly increased (*p* < 0.05) with the addition of egg white (M-TSPP+egg) and potato proteins (M-TSPP+potato) compared to M-TSPP. Potato and egg white proteins probably formed additional protein networks or improved interactions for gel formation compared to the other proteins, leading to increased structure formation. Studies on the impact of egg albumin on the thermal gelation of myofibrillar proteins are contradictory. Some authors concluded egg proteins caused disruption of the meat gel by interfering with the gelling process of the myofibrillar proteins or by the formation of mixed egg–myofibrillar protein gels, while others reported egg proteins participated in meat gel network formation [53]. Hunt, et al. [54] also observed a positive effect on gelation characteristics of Alaska pollock fish protein upon the addition of dried egg white protein. No significant difference in G’_76°C,batters_, G’_end,batter_, or δ_end,batter_ between M-TSPP and preparations with pea, gelatin, whey, blood plasma, or soy concentrate (M-TSPP+pea, M-TSPP+gelatin, M-TSPP+whey, M-TSPP+plasma, and M-TSPP+soy, respectively) could be observed. On the other hand, the studies of Wang, et al. [55] and Li, et al. [56] claimed an improvement of the gelling characteristics and structural strength of myofibrillar protein gels upon the addition of soy protein. Additionally, the addition of blood plasma has been shown to affect the thermal gelation of myofibrils and therefore influence the final gel strength [57,58]. Sun and Holley [48] stated that due to a lack of interaction between nonmeat and muscle proteins, it is possible that texture is negatively affected by interference with the gelation of the myofibrillar proteins. This could not be deducted from Figure 3, as the final gel strength (G’_end,batter_) of all phosphate-free batches with different proteins is similar or higher on average compared to M-TSPP.

The rather strong gelation potential of egg white and potato protein in the meat matrix during heating was also seen in the watery medium (Figure 1), as described in Section 3.1.1. Despite this similarity, batter parameters G’_76°C,batter_ and G’_end,batter_ were significantly higher for potato protein compared to egg white protein, which was not the case for G’_76°C, suspension_ and G’_end,suspension_ of the same proteins. Furthermore, blood plasma and whey protein resulted in similar values of G’_76°C,batter_ and G’_end,batter_ compared to egg white protein, which was not observed in G’_76°C, suspension_ and G’_end,suspension_ of the same proteins. In contrast, soy concentrate resulted in a significantly lower value of G’_76°C,batter_ compared to egg white protein, while this was not the case for G’_76°C,suspension._ On the other hand, G’_76°C,batter_ was lowest for preparations with pea protein and gelatin, which was identically reflected in G’_76°C,suspension_.

Evaluating these insights, it seems that the gelation potential of the different proteins, as determined in an aqueous medium during thermal processing (Section 3.1.1), was not always clearly noticeable in a meat system. This suggests the importance of a well-defined meat matrix, imitating industrial meat products, to determine and understand the impact of ingredient functionality. A food environment is a more complex system, where ingredients and other components (i.e., salts, lipids, and proteins) may interact, thus modifying the added value to the product quality of the functional ingredient.

#### 3.2.2. Emulsion Stability of Meat Batters and Cooking Loss of Cooked Sausages Influenced by Protein Source

Significant (*p* < 0.05) differences in emulsion stability and cooking loss (CL) were found between the different preparations (Table 1). Elimination of TSPP (M-TSPP) resulted in a significant increase of total expressible fluid (TEF) and thus lower emulsion stability, and increased CL compared to M+TSPP. These findings are in line with our former study [6]. It is known from the literature that TSPP is able to dissociate the actomyosin complex, releasing myosin, which can act as a natural emulsifier. Additionally, more myofibrillar proteins are extracted by TSPP, helping to stabilize the protein matrix in which water and fat are entrapped [1].

Preparations containing egg white protein (M-TSPP+egg), pea (M-TSPP+pea), soy concentrate (M-TSPP+soy), and blood plasma proteins (M-TSPP+plasma) significantly (*p* < 0.05) reduced TEF compared to M-TSPP and even resulted in similar percentages of TEF as the preparation containing phosphate (M+TSPP), indicating an equal stabilization of moisture and fat in the meat matrix. These proteins were thus able to compensate for the decreased emulsion stability due to phosphate elimination. On the other hand, there was no significant difference in TEF between M-TSPP and preparations with the addition of potato (M-TSPP+potato), whey proteins (M-TSPP+whey), and gelatin (M-TSPP+gelatin). Furthermore, the use of some proteins also caused a shift in composition (water vs. fat) of the drip loss. The relative amount of fat in the drip loss was significantly higher when adding blood plasma (M-TSPP+plasma), gelatin (M-TSPP+gelatin), soy concentrate (M-TSPP+soy), or whey protein (M-TSPP+whey) to phosphate-free sausages (M-TSPP). This could mean that fat stabilization in the meat matrix could be altered by using additional proteins, which could affect the final product characteristics such as texture or mouthfeel [59].

In almost all cases, CL significantly (*p* < 0.05) decreased with the addition of protein-based ingredients compared to M-TSPP. The addition of blood plasma (M-TSPP+plasma), whey proteins (M-TSPP+whey), egg white proteins (M-TSPP+egg), and soy concentrate (M-TSPP+soy) even resulted in similar CL as the cooked sausages containing phosphate (M+TSPP). Blood plasma proteins are good emulsifiers [9] and were found to be a useful substitute for polyphosphate in frankfurters, as they did not affect the water holding capacity and cooking losses compared to frankfurters containing 0.5% sodium tripolyphosphate [21]. Research by Prabhu [60] also indicated blood plasma was suitable to improve the emulsion stability, texture, flavor, and juiciness of comminuted meat products. Additionally, the use of pea protein (M-TSPP+pea) could significantly decrease CL compared to M-TSPP, although to a lesser extent than the previously mentioned proteins. On the other hand, the addition of gelatin (M-TSPP+gelatin) or potato protein (M-TSPP+potato) did not change CL compared to M-TSPP. This was in contrast with the study by Nieto, Castillo, Xiong, Álvarez, Payne and Garrido [20] in which cooking losses were reduced when 2.5% hydrolyzed potato proteins were added to phosphate-free meat emulsions.

Comparison between the intrinsic properties of the proteins discussed in Section 3.1 and their impact on fat and water binding characteristics of cooked sausages indicated limited analogy. Stronger gelation potential of the protein samples as measured in the watery medium would suggest better water and especially fat binding in meat products. Furthermore, proteins with good emulsifying capacities are expected to be able to stabilize emulsified meat products to a greater extent and contribute to reducing cooking loss (especially fat release). While potato and egg white protein both showed very good gelation properties, only the latter could positively improve the cooking yield. In contrast, pea proteins showed both low emulsifying capacity and gelling behavior in the watery medium, while in the cooked sausages, they could reduce cooking loss and TEF. Results even surpassed those of potato protein. Blood plasma proteins, showing an average gelation potential and good attribution to emulsion stability in the watery environment, outperformed the other proteins, with the exception of soy concentrate, regarding water and fat binding in cooked sausages. Again, these results underline the importance of a well-defined meat matrix, close to industrial meat products, to determine and fully understand the impact of ingredient functionality.

#### 3.2.3. pH of Cooked Sausages Influenced by Alternative Protein Source

Data (Table 1) showed that the elimination of TSPP (M-TSPP) resulted in significantly lower pH values compared to the model system containing TSPP (M+TSPP), which was in line with our former study [6]. The pH of TSPP (1% solution) is equal to 10.2 [1], which explains the pH difference between preparations M-TSPP and M+TSPP. Due to phosphate elimination, the pH of the meat product was decreased and was closer to the iso-electric pH of the myofibrillar proteins. This led to a reduction in their net charge and repulsion between proteins, causing a negative impact on water and fat binding, as seen in Section 3.2.2 [61]. The decrease in pH by phosphate elimination could not be compensated by the addition of protein-based ingredients, as seen in Table 1. Velemir, et al. [62] determined no significant difference in pH upon the addition of 1.5% whey or soy protein to sausages. Blood plasma, despite its higher pH, could also not remediate the lower pH of phosphate-free sausages, which was also seen in the research of Hurtado, Saguer, Toldrà, Parés and Carretero [21]. The proteins could therefore not contribute to water binding by generating a higher concentration of negative meat protein charges.

#### 3.2.4. Textural Properties of Cooked Sausages Influenced by Protein Type

The differences in hardness of the different preparations are limited (Table 1). Phosphate elimination (M-TSPP) did not significantly affect the hardness of the cooked sausages, which was in line with our former study [6]. A lower hardness might be expected when TSPP is eliminated, since TSPP dissociates the actomyosin complex, resulting in more proteins being available for emulsification and the formation of a more stable gel matrix during heating. Yet, gel strength at the end of thermal processing (see Section 3.2.1) also revealed no difference in G’_end,batter_ between M+TSPP and M-TSPP. On the other hand, the increase in CL when phosphate is eliminated could lead to a firmer meat product. The addition of gelatin (M-TSPP+gelatin) increased hardness compared to M-TSPP, despite no significant difference in CL being measured. Therefore, it could be concluded that gelatin itself had an impact on the final hardness of the phosphate-free cooked sausage, which could probably be attributed to its cold gelling properties [46]. The addition of the other protein-based ingredients did not significantly affect hardness compared to M-TSPP, but a significant increase in hardness compared to the reference sausage containing phosphate (M+TSPP) was determined upon the addition of pea, potato, egg white protein, and again gelatin. Nieto, Castillo, Xiong, Álvarez, Payne and Garrido [20] found that the addition of 2.5% hydrolyzed potato proteins had no effect on the hardness of phosphate-free frankfurters, which was also in line with our results. Furthermore, Youssef and Barbut [14] concluded that soy protein could increase or decrease the product texture depending on the type of soy used. The impact of whey proteins on hardness is linked to their degree of denaturation, which is dependent on their production process. In general, undenatured whey protein preparations deteriorate textural properties, while partially denatured whey concentrates enhance the binding and texture of sausages and other comminuted meat products [25]. This could possibly explain the mild impact on the observed hardness of whey proteins. Fernandez, et al. [63] also found no difference in hardness when 2% of dried egg white was added to chicken meat batters. Cofrades, Guerra, Carballo, Fernández-Martín and Colmenero [19] noted an increase in the product hardness of Bologna sausages when blood plasma was applied. This observation could not be established in this research.

## 4. Conclusions

The elimination of phosphate had a negative impact on several quality characteristics of cooked sausages. Next to an increase in cooking loss and reduced emulsion stability, a change in gel network formation during thermal processing could be observed, although the final gel strength was not influenced. The cause of these quality losses is mainly related to the reduced functionality of the myofibrillar proteins due to phosphate elimination. This research indicated that the addition of specific proteins could remediate the negative impact of phosphate elimination. However, it is important to keep in mind that different protein sources exhibit varying potential in this respect. Hereby, it is crucial to evaluate the potential of the proteins in a well-defined standardized meat matrix. The intrinsic protein properties, gelation and emulsification, related to improving meat quality are often evaluated in aqueous media. This study showed that protein characteristics determined in this manner did not entirely reflect their capacity to enhance the characteristics of phosphate-free emulsified meat products.

In phosphate-free cooked sausages, blood plasma and soy protein overall showed the most promising results, as no significant differences in terms of product hardness, cooking yield, or emulsion stability could be found compared to standard phosphate-containing sausages. These proteins may therefore provide an added value for the meat industry to further reduce E-numbers and contribute to the healthy image of meat products. Other screened proteins, such as egg white, pea, and whey protein, also proved to be beneficial, yet the quality level of the phosphate-containing sausages could not be equaled. Potato protein and gelatin showed the least improvement to the phosphate-free cooked sausages.

Future research can be conducted on the use of combinations of different protein sources or mixtures of proteins with certain hydrocolloids to further remediate the loss of quality due to phosphate elimination in emulsified meat products.

## Figures and Tables

**Table 1:** Structural parameters of sausage batters (stress sweeps) and hardness, pH, water, and fat binding characteristics (CL, TEF, and relative amount of H_2_O) of cooked sausages with phosphate (M+TSPP), without phosphate (M-TSPP), and several protein-based ingredients instead of phosphate (M-TSPP+protein). Mean values and standard deviations are presented (n = 2). Different letters indicate significant differences at *p* < 0.05. Abbreviations used: M (Model), TSPP (tetrasodium pyrophosphate), LVR_batter_ (linear viscoelastic region of the sausage batter), G*_batter_ (complex modulus of the sausage batter), δ_batter_ (phase angle of the sausage batter), TEF (total expressible fluid), and CL (cooking loss). Superscripts a–e: different letters indicate significant differences (*p* < 0.05) between different treatments.

	M+TSPP	M-TSPP	M-TSPP +egg	M-TSPP +pea	M-TSPP +potato	M-TSPP +soy	M-TSPP +plasma	M-TSPP +gelatin	M-TSPP +whey
**Stress sweeps**									
LVR_batter_/Pa	25 ± 4 ^bc^	56 ± 9 ^a^	7 ± 7 ^c^	53 ± 8 ^a^	45 ± 17 ^a,b^	54 ± 5 ^a^	48 ± 4 ^a^	56 ± 6 ^a^	42 ± 0 ^a,b^
G*_batter_/10^2^Pa	12 ± 1 ^d^	36 ± 0.1 ^c^	227 ± 67 ^a^	46 ± 2 ^c^	38 ± 2 ^c^	52 ± 0.6 ^b,c^	39 ± 4 ^c^	77 ± 5 ^b^	45 ± 1 ^c^
*δ_batter_*/°	37.2 ± 0.1 ^a^	11.3 ± 0.2 ^c,d^	11.2 ± 1.3 ^c,d^	11.1 ± 0.0 ^c,d^	12.0 ± 0.0 ^b,c^	10.8 ± 0.2 ^d^	10.7 ± 0.3 ^d,e^	9.8 ± 0.2 ^e^	12.8 ± 0.5 ^b^
**Stability**									
TEF/%	1.9 ± 0.4 ^c^	5.2 ± 0.6 ^a^	3.2 ± 0.6 ^b,c^	2.9 ± 0.5 ^c^	4.8 ± 0.7 ^a,b^	1.9 ± 0.6 ^c^	2.5 ± 0.6 ^c^	6.5 ± 0.7 ^a^	5.4 ± 0.3 ^a^
Relative amount of H_2_O/%	89.1 ± 1.2 ^a,b,c^	90.8 ± 0.4 ^a,b^	89.2 ± 1.0 ^a,b,c^	88.8 ± 0.3 ^a,b,c^	91.8 ± 0.3 ^a^	85.7 ± 1.1 ^c,d^	86.1 ± 1.5 ^c,d^	87.5 ± 0.1 ^b,c,d^	84.1 ± 1.3 ^d^
CL/%	0.6 ± 0.2 ^d^	5.7 ± 0.6 ^a^	2.1 ± 0.2 ^c,d^	3.2 ± 0.1 ^b,c^	4.2 ± 0.1 ^a,b^	1.9 ± 0.4 ^c,d^	0.9 ± 0.1 ^d^	5.4 ± 1.0 ^a^	1.2 ± 0.1 ^d^
**pH**/(-)	6.98 ± 0.09 ^a^	6.62 ± 0.00 ^b,c^	6.59 ± 0.01 ^b,c^	6.52 ± 0.07 ^b,c^	6.46 ± 0.07 ^c^	6.44 ± 0.04 ^c^	6.69 ± 0.08 ^b^	6.41 ± 0.04 ^c^	6.43 ± 0.01 ^c^
**Hardness**/N	4.3 ± 0.0 ^c^	4.8 ± 0.1 ^b,c^	5.6 ± 0.4 ^a,b^	5.3 ± 0.2 ^a,b^	5.2 ± 0.1 ^a,b^	5.0 ± 0.4 ^a,b,c^	5.0 ± 0.2 ^a,b,c^	5.8 ± 0.2 ^a^	5.1 ± 0.2 ^a,b,c^

**Figure A1:**
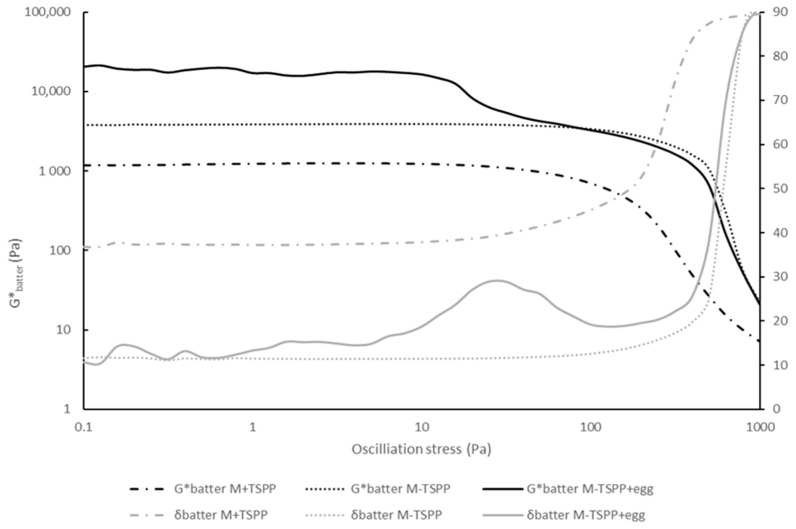
Stress sweep curves of meat batters containing phosphate (M+TSPP, dash-dot line), without phosphate (M-TSPP, dotted line), and without phosphate-containing egg white proteins (M-TSPP+egg, full line). The complex modulus (G*, black color) and phase angle (δ, grey color) are displayed on the primary and secondary y-axis, respectively. Confidence intervals are not included to enhance the readability of the graph (n = 2).

## Data Availability

Not applicable.

## Appendix A

Raw phosphate-free sausage batter containing 2% egg white protein (M-TSPP+egg) gave rise to a significantly lower LVR_batter_, and, at the same time, a remarkably high G*_batter_ compared to M-TSPP, as seen in Table 1 (main text). In order to gain insight into this striking observation, the stress sweeps as seen in Figure A1 were evaluated.

According to Glorieux, Goemaere, Steen and Fraeye [6], the LVR is calculated as the stress level at which G* deviates more than 5% from a constant G* (plateau) value and indicates irreversible structure breakdown. However, the raw sausage batter containing egg white proteins (M-TSPP+egg white) contained two plateau regions in which G’ and G” (and thus G*) were independent of the applied stress amplitude. The first plateau was characterized by a high G_batter_* value and ranged up to ±10 Pa, the stress value at which structure breakdown occurred. However, from around a stress value of 30 Pa, the structure stabilized again, resulting in another plateau that reached stress values of ±200 Pa until irreversible structure breakdown occurred (Figure A1). The two LVR regions could possibly be explained by the presence of two distinct protein structures. The first plateau is characterized by G*_batter_ higher than G*_batter_ of M-TSPP, which may be attributed to the presence of the egg white proteins. The second plateau is characterized by G*_batter_ values in the same order of magnitude as M-TSPP, and the LVR ends at a comparable stress value, presumably indicating that this part of the LVR was stabilized independently of the added protein-based ingredient.
